# Relevance of Liquid-Liquid Phase Separation of Supersaturated Solution in Oral Absorption of Albendazole from Amorphous Solid Dispersions

**DOI:** 10.3390/pharmaceutics13020220

**Published:** 2021-02-05

**Authors:** Kyosuke Suzuki, Kohsaku Kawakami, Masafumi Fukiage, Michinori Oikawa, Yohei Nishida, Maki Matsuda, Takuya Fujita

**Affiliations:** 1 Pharmaceutical and ADMET Research Department, Daiichi Sankyo RD Novare Co., Ltd., 1-16-13, Kitakasai, Edogawa-ku, Tokyo 134-8630, Japan; 2 Research Center for Functionals Materials, National Institute for Materials Science, 1-1 Namiki, Tsukuba, Ibaraki 305-0044, Japan; 3 Pharmaceutical R&D, Ono Pharmaceutical Co., Ltd., 3-3-1, Sakurai, Shimamoto-cho, Mishima-gun, Osaka 618-8585, Japan; fukiage@ono.co.jp; 4 Pharmaceutical Development Department, Sawai Pharmaceutical Co., Ltd., 5-2-30, Miyahara, Yodogawa-ku, Osaka 532-0003, Japan; m.oikawa@sawai.co.jp; 5 Technology Research & Development, Sumitomo Dainippon Pharma Co., Ltd., 33-94, Enoki-cho, Suita, Osaka 564-0053, Japan; yohei-nishida@ds-pharma.co.jp; 6 Research & Development Division, Towa Pharmaceutical Co., Ltd., 134, Chudoji Minami-machi, Shimogyo-ku, Kyoto 600-8813, Japan; mk-matsuda@towayakuhin.co.jp; 7 College of Pharmaceutical Sciences, Ritsumeikan University, 1-1-1 Noji-Higashi, Kusatsu, Shiga 525-8577, Japan; fujita-t@ph.ritsumei.ac.jp

**Keywords:** amorphous solid dispersions, albendazole, oral absorption, liquid-liquid phase separation, dissolution study, supersaturation

## Abstract

Amorphous solid dispersion (ASD) is one of the most promising formulation technologies for improving the oral absorption of poorly soluble drugs, where the maintenance of supersaturation plays a key role in enhancing the absorption process. However, quantitative prediction of oral absorption from ASDs is still difficult. Supersaturated solutions can cause liquid-liquid phase separation through the spinodal decomposition mechanism, which must be adequately comprehended to understand the oral absorption of drugs quantitatively. In this study, albendazole (ALZ) was formulated into ASDs using three types of polymers, poly(methacrylic acid-*co*-methyl methacrylate) (Eudragit) L100, Vinylpyrrolidone-vinyl acetate copolymer (PVPVA), and hydroxypropyl methylcellulose acetate succinate (HPMCAS). The oral absorption of ALZ in rats administered as ASD suspensions was not explained by dissolution study but was predicted using liquid-liquid phase separation concentration, which suggested that the absorption of ALZ was solubility-limited. The oral administration study in dogs performed using solid capsules demonstrated the low efficacy of ASDs because the absorption was likely to be limited by dissolution rate, which indicated the importance of designing the final dosage form of the ASDs.

## 1. Introduction

Candidate compounds developed in the pharmaceutical industry frequently exhibit extremely low aqueous solubility and may be poorly absorbed after oral administration, which is one of the major issues in drug development. For such compounds, supersaturating formulation technologies are often used to improve their oral absorption [1,2,3]. The method for predicting drugs’ oral absorption from supersaturating formulations using dissolution studies is still under debate [4]. Specifically, non-sink conditions must be used in the analysis because supersaturation must be investigated during the dissolution process [5]. However, the dissolution pattern of compounds in supersaturating formulations is significantly influenced by supersaturation degree [6,7]. The pH shift from acidic to neutral conditions is worth being tested, especially when the drugs, excipients, or both have pH-dependent dissolution properties [8]. The absorption sink’s role also needs to be considered because it changes the degree of supersaturation [6]. The effects of surface-active agents, including drug solubilization [9,10] and acceleration of crystallization [8], have complicated effects on the supersaturating dissolution behavior, which must also be considered. On the other hand, the dissolution test should be simple to ensure intra-individual reproducibility.

Amorphous solid dispersion (ASD) is one of the most promising techniques for improving poorly soluble candidates’ solubility. Dissolution profiles of ASDs are quite different from conventional formulations using crystalline drugs, characterized by supersaturation and a gradual decrease in concentration [1,11]. During this process, the supersaturated solution may cause liquid-liquid phase separation (LLPS) based on the spinodal decomposition mechanism [11,12]. This process produces highly concentrated (nano)droplets/particles, which cannot be absorbed directly but may act as drug reservoirs, shuttles, or both to carry drug molecules effectively in the mucus layer [9,13,14,15]. The drug concentration in the continuum phase (i.e., LLPS concentration) was relevant to oral absorption [4,8]. Thus, it is critical to understand and control LLPS after the dissolution of ASDs to use the ASD technology effectively.

**Figure 1:**
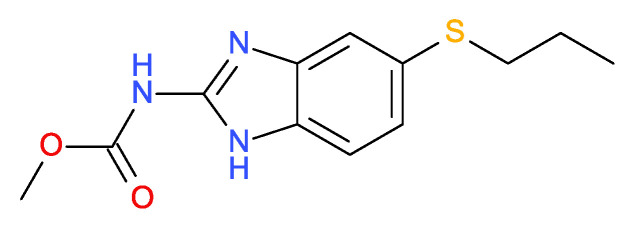
Chemical structure of ALZ.

Albendazole (ALZ) is a weak basic compound with low aqueous solubility (Figure 1, Table 1) [16,17,18]. Some variability can be found for its reported intrinsic solubility, but it is typically below 1 μg/mL, which is slightly improved in fasted-state simulated intestinal fluid (FaSSIF) and significantly enhanced in fed-state simulated intestinal fluid (FeSSIF). Although some successful attempts to apply ASD to ALZ to improve its oral absorption have already been reported, the detailed mechanism of the enhanced oral absorption has not yet been elucidated. Kohri et al. [19] showed that an ASD of ALZ with hydroxypropyl methylcellulose (HPMC) and HPMC phthalate significantly improved the dissolution properties and maintained supersaturation for >8 h. In the oral administration study of an ALZ ASD in rabbits with low gastric acidity, the area under the concentration-time curve (AUC) was three-fold higher than that of the physical mixture. Despite the successful application of ASD in this animal study, no insights about the formulation strategy were obtained because only one ASD was investigated in their study. Silvina et al. [20] found that the AUC after oral administration of an ALZ ASD to mice using Pluronic 188 was much higher than that that after administration of its crystalline suspension [20]. The ASD exhibited a superior dissolution rate compared to that of the crystalline ALZ. In this case, the improvement in dissolution behavior and oral absorption was mainly attributed to the polymer’s surfactant-like properties, including enhancement of wettability and the micellar solubilization effect.

Earlier studies of ALZ ASDs did not recognize the occurrence of LLPS during dissolution. In this study, the LLPS behavior of supersaturated ALZ solutions was investigated, and then an oral administration study of the ASDs was conducted in rats and dogs to observe the relevance of LLPS behavior to oral absorption.

## 2. Materials and Methods

### 2.1. Chemicals

ALZ was purchased from Sigma-Aldrich (St. Louis, MO, USA) and was ground using a mortar and pestle before use. Poly(methacrylic acid-*co*-methyl methacrylate) (Eudragit L100), hydroxypropyl methylcellulose acetate succinate (HPMCAS), and vinylpyrrolidone-vinyl acetate copolymer (Kollidon VA64, PVPVA) were obtained from Evonik Industries AG (Essen, Germany), Shin-Etsu Chemical Co., Ltd. (Tokyo, Japan), and BASF SE (Ludwigshafen am Rhein, Germany), respectively. Mannitol was supplied by Nacalai Tesque, Inc. (Kyoto, Japan). All chemicals were used as supplied.

### 2.2. Physical Characterization

Powder X-ray diffraction (PXRD) measurements were performed using a D8 DISCOVER with a general area detector diffraction system (Bruker, MA, USA) using CuKα radiation. The tube voltage and current were 40 kV and 40 mA, respectively. Data were collected in the range of 5° to 40° with intervals of 0.02° (2 theta), and the scan speed was 5°/min.

Simultaneous measurement of thermogravimetric (TG) and differential thermal analysis (DTA) was performed on TG/DTA 6200 (Hitachi High-Tech, Tokyo, Japan) with a scan rate of 10 °C/min. The temperature was calibrated with indium. Approximately 2 mg of the sample (as an ALZ equivalent) was evaluated using an aluminum open pan. Dry nitrogen was used as an inert gas at a flow rate of 100 mL/min.

The formulation morphology was examined using scanning electron microscopy (SEM) with the SU8000 SEM (Hitachi, Tokyo, Japan) at an accelerating voltage of 1 kV. Samples were loaded on carbon tapes under nitrogen flow to avoid moisture adsorption, followed by sputter coating using a platinum coater (E-1030 ion sputter, Hitachi) before analysis.

### 2.3. Solubility Measurement of ALZ

Approximately 10 mg of crystalline ALZ was loaded in a test tube (*n* = 3), to which 5 mL of phosphate buffer (PB, 50 mM, pH 7) was added in the presence or absence of the polymer (0.1 *w/v*%). The solutions were then lightly sonicated, vortexed, and then rotated at approximately 50 rpm at 25 or 37 °C for 1 day. Each solution was filtered using a nylon syringe filter with a pore size of 0.2 μm (Sanplatec, Osaka, Japan). For the measurement of solubility at 37 °C, the syringes and syringe filters were warmed in an oven at 37 °C prior to use. The filtrate was diluted with ethanol and measured using a high-performance liquid chromatography (HPLC) system equipped with an octadecylsilyl (ODS) YMC-Pack Pro C18 column (150 mm × 2.0 mm ID, YMC, Kyoto, Japan). A water/acetonitrile mixture at a ratio of 55:45 was used as the mobile phase at a flow rate of 0.2 mL/min. The injection volume and the column temperature were 2 mL and 30 °C, respectively. Measurements were made at a concentration range from 0.1 to 20 μg/mL at a wavelength of 295 nm using a photodiode array detector, where the linearity was confirmed.

### 2.4. Preparation of ASDs of ALZ

ALZ and the polymer were dissolved separately in 1,4-dioxane at concentrations of 10 and 20 mg/mL, respectively, and then the solutions were mixed to achieve an ALZ:polymer ratio of 1:3 (*w/w*). For the PVPVA, a solution with an ALZ:polymer ratio of 1:4 was also prepared. The solutions were frozen using liquid nitrogen and then freeze-dried using the VirTis Advantage EL freeze dryer (Warminster, PA, USA). The shelf was initially maintained at −50 °C, followed by successive drying at −20 °C, −5 °C, and 10 °C for 10 h each. Finally, the residual solvents were removed at 35 °C under vacuum, and each ASD was stored in a refrigerator before use. An equal amount of crystalline ALZ and mannitol were mixed using a mortar and pestle to prepare a physical mixture as the control sample used for the dissolution and administration studies. All the formulations were confirmed to be physically and chemically stable during the storage (for at least three months).

### 2.5. LLPS Concentration and Particle Properties

An appropriate amount of a dimethylacetamide solution of ALZ was added to 50 mL PB in the polymer’s presence or absence (0.1 *w/v*%). The solutions were stirred at approximately 200 rpm at 25 °C. The turbidity was measured at a wavelength of 500 nm using a DU-800 spectrophotometer (Beckman Coulter, Brea, CA, USA) after stirring for 30 min. The phase separation concentration was estimated from the breakpoint of the turbidity-concentration curves [7,8]. The suspensions’ particle size and zeta potential were measured using a Zetasizer Nano ZS (Malvern Panalytical, Malvern, UK). All measurements were repeated three times, and the mean particle size was determined using the cumulant method.

LLPS behavior was also observed using ASD as a starting material. ASD or crystalline ALZ (as a physical mixture with mannitol) was dispersed at a concentration of 40 µg/mL as the ALZ equivalent in 50 mL PB at 25 °C while stirring at approximately 200 rpm in beakers. Solutions were sampled over time and immediately filtered through syringe filters with a pore size of 0.2 or 0.7 µm (Millex-LG, Merck Millipore, Burlington, MA, USA and GF/F, Whatman, Buckinghamshire, UK, respectively). The ALZ concentration of the filtered solutions was determined using HPLC as described above. The measurements were duplicated to obtain averaged values. This observation is described as the LLPS dissolution test hereafter.

### 2.6. µDISS Dissolution Study

The dissolution study was performed using a μDISS Profiler™ system (pION Inc., Billerica, MA, USA). The first and second fluids of the Japanese Pharmacopeia (JP1 and JP2, respectively) were used as dissolution media. The μDISS vessels were placed in a thermostatic chamber at 37 °C, 10 mL of JP1 (pH 1.2) or JP2 (pH 6.8) was added to the vessel, and then the UV probe was immersed in the solution. The ASD or crystalline ALZ was dispersed at a concentration of 15 µg/mL as the ALZ equivalent while stirring at 200 rpm, and the UV spectra were acquired over time to measure the ALZ concentration.

### 2.7. Oral Administration Study in Rats

All experiments using rats were approved by the Ethical Review Committee of DaiichiSankyo RD Novare (Exp. No. 2017-024, 2017-027 (2017)). Male Crl:CD(SD) rats (6–7-week-old, Charles River Laboratories Japan, Yokohama, Japan) were housed in a temperature-controlled room at 23 ± 2 °C with a relative humidity of 55 ± 20%, and a 12 h light/dark cycle. The rats were starved for 16 h before and 6 h after administration with free access to water. Formulations were dispersed in 0.5% methylcellulose (MC) (for crystalline ALZ) or purified water (for ASDs) using a homogenizer, followed by immediate administration to rats (*n* = 3) at a dose of 10 mg/10 mL/kg as the ALZ equivalent. Approximately 150 μL blood samples were withdrawn through the jugular vein using pre-heparinized syringes 0.25, 0.5, 1, 2, 4, and 8 h after administration. Plasma samples were prepared by centrifuging the blood at 3000× *g* for 3 min and were then stored at −20 °C until the analysis. 

Plasma concentration of ALZ sulfoxide, a metabolite of ALZ, was measured using a liquid chromatography-tandem mass spectrometry (LC-MS/MS) method (API-4000, SCIEX UPLC, Waters, Milford, MA, USA). The detailed procedure was described elsewhere [7]. Briefly, the plasma sample (20 µL) was mixed with a 50% (*v/v*) aqueous acetonitrile solution, to which 200 μL of acetonitrile/methanol (75/25 (*v/v*)) was added. Niflumic acid was used as the internal standard. The mixture was filtered and measured. The linearity was confirmed in a concentration range of 0.005–5 μg/mL.

### 2.8. Oral Administration Study in Dogs

All experiments in the beagle dogs were approved by the Ethical Review Committee of KAC Co. (Exp. No. 17-1220 (2017)). Male beagle dogs (body weight: 10.8–13.2 kg, Kitayama Labes, Ina, Japan) were housed in a temperature-controlled room at 23–27 °C under a 12 h light/dark cycle. The dogs were starved 16 h before and for 4 h after administration and allowed free access to water. Each formulation was loaded into #00 gelatin capsules, except for the HPMCAS ASD, for which aqueous suspension was also prepared, and then administered to the dogs (*n* = 3) at a dose of 10 mg/0.5 mL/kg. Pentagastrin was administered intramuscularly at a dose of 10 μg/kg 30 min before and 30/90 min after administration. Approximately 2.5 mL blood samples were withdrawn using pre-heparinized syringes 0.25, 0.5, 1, 2, 4, and 7 h after administration, centrifuged at 1800× *g* for 10 min and then stored at −80 °C until the analysis.

The plasma ALZ sulfoxide concentration was measured using LC-MS/MS (Ultimate 3000 Rapid Separation LC, Q Exactive, Thermo Scientific, Waltham, MA, USA). The plasma samples were processed in the same manner as done for the rat study with phenacetin as the internal standard. The mixture was filtered and subjected to measurements. Linearity was confirmed in a concentration range of 0.0003–1 μg/mL.

## 3. Results

### 3.1. Physicochemical Properties of Crystalline ALZ and ASDs

**Figure 2:**
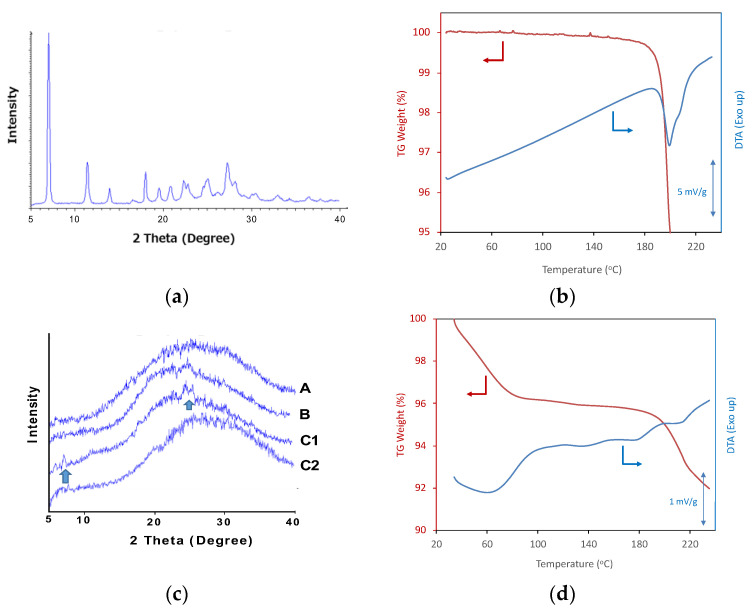
(**a**) PXRD pattern of crystalline ALZ. (**b**) TG-DTA curve of crystalline ALZ. (**c**) PXRD patterns of ALZ ASDs. A: Eudragit/ALZ = 3/1, B: HPMCAS/ALZ = 3/1, C1: PVPVA/ALZ = 3/1, C2: PVPVA/ALZ = 4/1. (**d**) TG-DTA curve of PVPVA/ALZ = 3/1 ASD.

**Figure 3:**
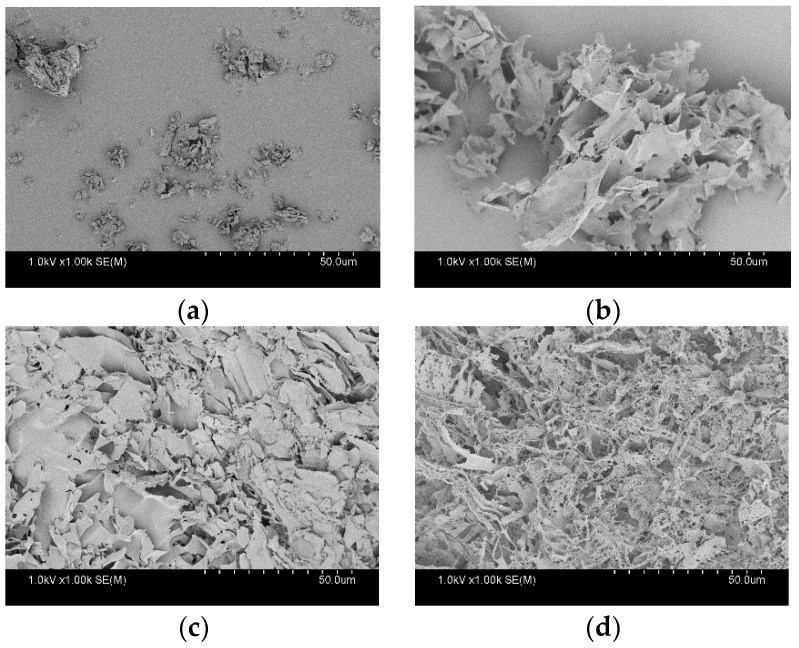
SEM images of (**a**) crystalline ALZ, (**b**) HPMCAS ASD, (**c**) Eudragit ASD, and (**d**) PVPVA ASD.

The PXRD and TG-DTA patterns of crystalline ALZ are presented in Figure 2a,b, respectively. Sharp diffraction peaks were confirmed in the PXRD pattern. An endothermic melting peak was found at 199 °C in the DTA curve; however, thermal degradation was also initiated approximately at the same temperature. Thus, thermal analysis was not an appropriate method for analyzing the crystalline property of ALZ because of the overlapping of melting and degradation behaviors. No crystalline diffraction peaks were observed for the ASDs prepared using Eudragit or HPMCAS (Figure 2c), whereas the PVPVA ASD showed small diffraction peaks at an ALZ:polymer ratio of 1:3. ALZ Crystals were found only for this ASD in polarized microscopy (PLM) analysis (supplementary material). The diffraction peak disappeared when the PVPVA content was increased to a ratio of 1:4 (Figure 2c), which was also confirmed in the PLM image (supplementary material). Thus, this ratio was employed only for PVPVA in the following studies. Figure 2d shows the TG-DTA pattern of PVPVA/ALZ = 3/1 ASD. Although a small broad endothermic peak was found at the melting temperature in the DTA curve, it was difficult to assign this event as the melting because of decrease of the weight at the same temperature that indicated degradation. Thus, thermal analysis was not reliable for finding a small amount of crystalline ALZ. Nevertheless, no melting behaviors were observed for all ASDs (supplementary material). Absence of ALZ crystals was also confirmed by the PLM analysis (supplementary material). Figure 3 shows SEM images of the crystalline ALZ and its ASDs. The size of the ALZ crystals was confirmed to be reduced to a micrometer order after grinding. A porous structure was confirmed for all the ASDs, which is typical for freeze-dried ASDs, and no crystalline particles were found.

### 3.2. Dissolution and LLPS Properties of ALZ ASDs

Table 2 shows the crystalline ALZ’s equilibrium solubility, LLPS concentrations, and particle properties above the LLPS concentrations. The poor solubility in PB was slightly improved in the presence of Eudragit and HPMCAS, and the solubilities did not depend significantly on temperature under these conditions. The pH did not change after equilibration in all cases. In the absence of the polymer, LLPS occurred at 1.4 μg/mL, which was >10-fold the solubility, whereas it increased in the presence of polymers in the order of 7.2 μg/mL, 7.0 μg/mL, and 3.8 μg/mL for PVPVA, HPMCAS, and Eudragit, respectively. The mean particle size found in each solution was >1 μm, except for the HPMCAS solution, ca. 0.22 μm.

The size distribution of the particles found in the Eudragit solution was broad, although they exhibited the highest zeta potential value. In the polarized microscopy analysis, crystal particles were found in all samples except for HPMCAS (data not shown). Thus, the observed particle sizes were likely not of the LLPS particles but were of ALZ crystals. Crystallization was not evident in the HPMCAS solution because the particle size was too small in the microscopic investigation. The zeta potentials were highly negative in the presence of Eudragit and slightly negative in the presence of HPMCAS, which is a typical observation [7,8].

**Figure 4:**
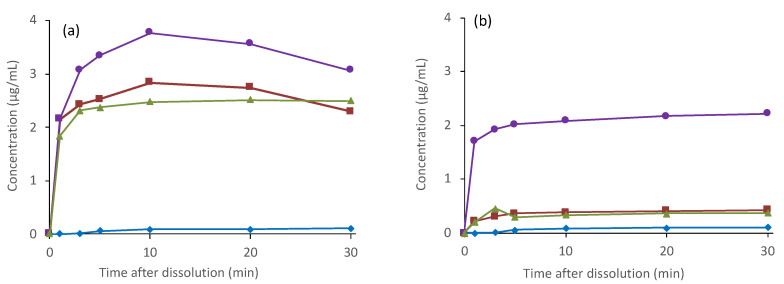
Concentration profiles of ALZ eluted from crystalline ALZ and ALZ ASDs in the LLPS dissolution test. Filtration was performed using syringe filters with pore sizes of (**a**) 0.7 μm or (**b**) 0.2 μm. Symbols: Crystalline ALZ (◆), PVPVA ASD (■), HPMCAS ASD (▲), Eudragit ASD (●).

Figure 4a shows the LLPS dissolution test results, where suspensions were treated using syringe filters with a pore size of 0.7 μm. ALZ concentrations in the eluate from the crystalline ALZ did not exceed 0.15 μg/mL, which was almost explainable from its equilibrium solubility in PB. In contrast, ALZ concentrations reached much higher values when ASDs were subjected to the study. The Eudragit ASD showed the highest concentration of ALZ, followed by the PVPVA and HPMCAS ASDs. The observed concentrations of these ASDs were higher than the equilibrium solubility in the polymers’ presence (Table 2). The concentrations of the Eudragit and PVPVA ASDs started to decrease from 10 min, indicating that precipitation was induced by excess solids or crystallization of ALZ or both.

The LLPS dissolution tests’ filtrates using filters with 0.7 μm pore size had a slightly turbid appearance, which suggested that small particles permeated through the syringe filters. This is especially expected for HPMCAS ASD because it creates small LLPS particles (Table 2). Therefore, the experiments were repeated using membrane filters with a 0.2 μm pore size (Figure 4b). All the filtrates in this experiment were transparent. The ALZ concentrations of the HPMCAS and PVPVA ASDs decreased dramatically, indicating the presence of particles with sizes between 0.2 and 0.7 μm. The concentration of Eudragit ASD also decreased by approximately 1 μg/mL.

**Figure 5:**
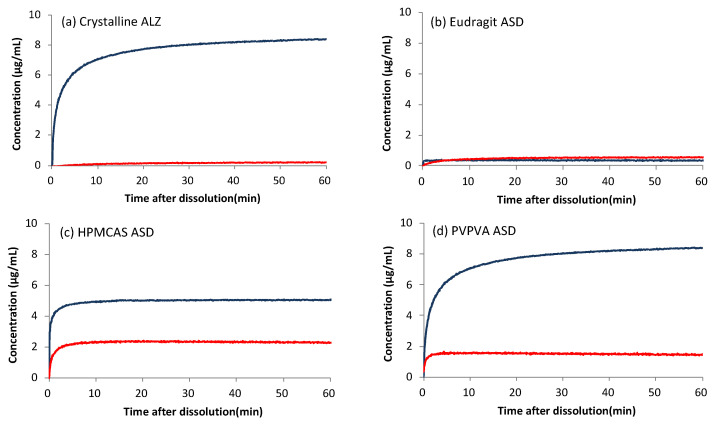
μDiss dissolution test of (**a**) crystalline ALZ and (**b**–**d**) ALZ ASDs. Types of polymers are indicated in the figure. Blue and red lines represent dissolution profiles in JP1 (pH 1.2) and JP2 (pH 6.8) solutions.

Although the ALZ concentration after filtration with a 0.2 μm filter in the LLPS dissolution test could be expected to be equal to that of the LLPS, it was much lower. One possible explanation is enhanced precipitation induced by excess solids [7]. However, the ALZ concentrations from the HPMCAS and PVPVA ASDs agreed well with the equilibrium solubility, which was likely attributable to the crystallization of ALZ. The dissolution patterns supported this hypothesis for the Eudragit and PVPVA ASDs in Figure 4a. Crystallization was not suspected for the HPMCAS ASD from its dissolution pattern (Figure 4a). The crystals might pass through the syringe filters because of the smallness of the particle size. Figure 5 shows the dissolution properties of crystalline ALZ and its ASDs evaluated using the μDISS apparatus, where the order of the achieved concentrations did not agree with that of the equilibrium solubility. In the acidic environment, the best dissolution was observed with the crystalline ALZ and PVPVA ASD, followed by HPMCAS ASD and Eudragit ASD. None of these formulations reached equilibrium solubility, which was reported to be 184 μg/mL (Table 1), presumably because the observation was made only for 1 h. The poor dissolution behavior of ALZ from Eudragit and HPMCAS ASDs could be attributable to these acidic polymers’ low solubility. Elution of ALZ was likely to be disturbed in the presence of these insoluble polymers.

In contrast, the dissolution under neutral pH conditions was enhanced the most by HPMCAS ASD, followed by PVPVA ASD. The concentrations achieved with these ASDs was higher than that of the equilibrium solubility (Table 2), indicating that supersaturation was attained. Eudragit ASD also slightly improved the dissolution, where the ALZ concentration almost agreed with the equilibrium solubility. Crystalline ALZ was rarely dissolved under the neutral pH condition.

### 3.3. Oral Administration Study

**Figure 6:**
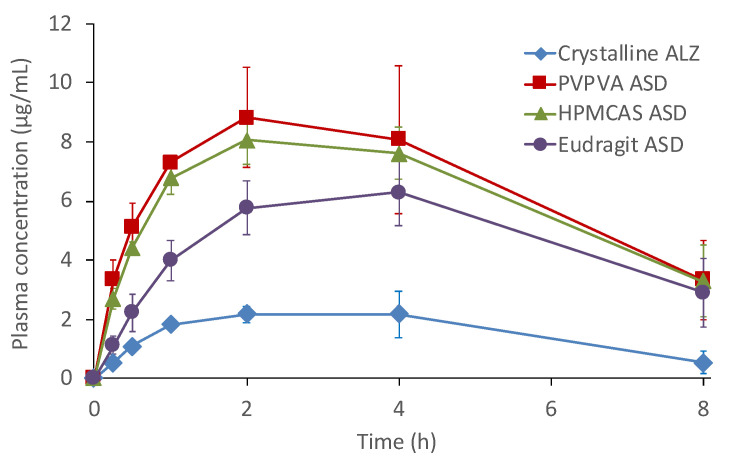
Plasma ALZ sulfoxide concentration profiles after oral administration of crystalline ALZ and its ASDs in rats at a dose of 10 mg/kg (*n* = 3). Symbols: Crystalline ALZ (◆), PVPVA ASD (■), HPMCAS ASD (▲), Eudragit ASD (●).

Figure 6 shows the results of the oral absorption study of crystalline ALZ and its ASDs in rats. The absorption was considerably higher with the ASDs relative to crystalline ALZ. The pharmacokinetic (PK) parameters are shown in Table 3. The PVPVA and HPMCAS ASDs showed almost the same plasma concentration profiles with AUC values 4-fold higher than that of the crystalline ALZ. The Eudragit ASD also improved the oral absorption; however, the AUC was slightly lower than those of the other two ASDs, which was approximately 3-fold of the crystalline ALZ.

**Figure 7:**
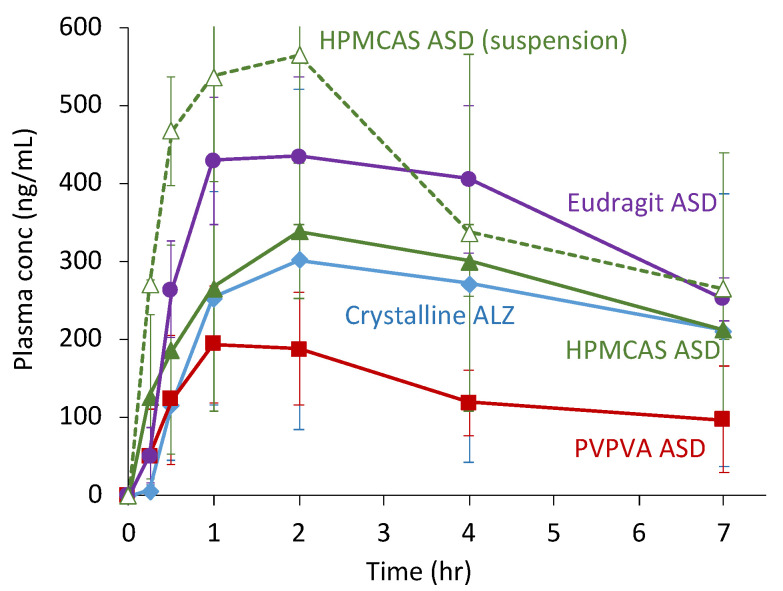
Plasma ALZ sulfoxide concentration profiles after oral administration of crystalline ALZ and its ASDs in beagle dogs at a dose of 10 mg/kg (*n* = 3). Formulations were administered as capsules unless otherwise noted as suspension. Symbols: crystalline ALZ (◆), PVPVA ASD (■), HPMCAS ASD (▲), HPMCAS ASD (suspension) (△), Eudragit ASD (●).

Figure 7 and Table 4 show the oral absorption study results of the crystalline ALZ and its ASDs in beagle dogs. Administration of HPMCAS ASD as a suspension considerably enhanced the oral absorption, similar to the rat study’s findings. However, the absorption behavior was totally different when the same ASD was administered as capsules, where the ASDs were filled as solid forms. The absorption from the HPMCAS ASD was comparable to the crystalline ALZ, which was also administered as capsules. That from the Eudragit ASD was higher than that from the HPMCAS ASD, whereas that from the PVPVA ASD was worse than that from crystalline ALZ.

## 4. Discussion

### 4.1. Dissolution and LLPS Behaviors of ALZ and Its ASDs

The LLPS concentration increased with the polymer’s addition, and the highest value was observed with PVPVA, followed by HPMCAS and Eudragit. If the crystallization tendency of a drug is low, LLPS is not influenced by the presence of polymers [8]. Thus, this observation indicates that these are values of “apparent” LLPS, which is influenced by the crystallization of ALZ [7,8]. The occurrence of crystallization was also obvious from concentration profiles during the LLPS dissolution study (Figure 4a). The “apparent” LLPS was shown to be a good predictor of the AUC of oral absorption controlled by solubility [8]. The size of the LLPS particles may also be a factor that influences absorption from ASDs. The particle size measurement based on the light scattering principle is a well-established methodology; however, the data analysis can frequently be misleading, especially when the particles have wide size distribution. Thus, we evaluated the particle size from the dissolution study, where syringe filters with multiple pore sizes were used. It provided direct information on the fraction of the particles in specific particle size ranges. Also, it enabled discussion on the crystallization of ALZ during the LLPS behavior.

The μDISS dissolution study showed no dissolution advantage for the ASDs under the acidic pH condition, whereas the dissolution was enhanced by formulating the drug with HPMCAS or PVPVA under the neutral pH condition. The observed concentrations were well below LLPS concentrations but higher than the equilibrium solubilities. Although the reason for the effective maintenance of supersaturation in the μDISS dissolution study relative to the LLPS dissolution in beakers is unclear, it may have been due to the test vessels’ smaller scale. As ALZ was not charged under the neutral pH condition, the supersaturated state was likely stabilized by interaction with polymers, including hydrophobic interaction and hydrogen bonding. In the μDISS dissolution study, the ALZ concentration was measured using a UV probe, which is expected to detect only dissolved ALZ. However, in the LLPS dissolution study, where the filtrate’s ALZ concentration was determined, totally different dissolution profiles were obtained. The dissolution appeared to be improved with the ASDs, and Eudragit appeared to be the most effective polymer. However, the ALZ concentration was affected by the pore size of the syringe filters, which indicated particles’ presence in the filtrate.

Moreover, the particles were not likely to be the result of LLPS, but were more likely caused by crystallization. The highest concentration of the Eudragit ASD after filtration with a 0.2 μm filter can be explained by the particles’ wide size distribution, as expected from its large PDI. The LLPS particles’ size was likely to be maintained only in the HPMC solution even after crystallization. These findings are important for understanding the oral absorption results.

### 4.2. Relationship between Dissolution Behavior and Oral Absorption

**Figure 8:**
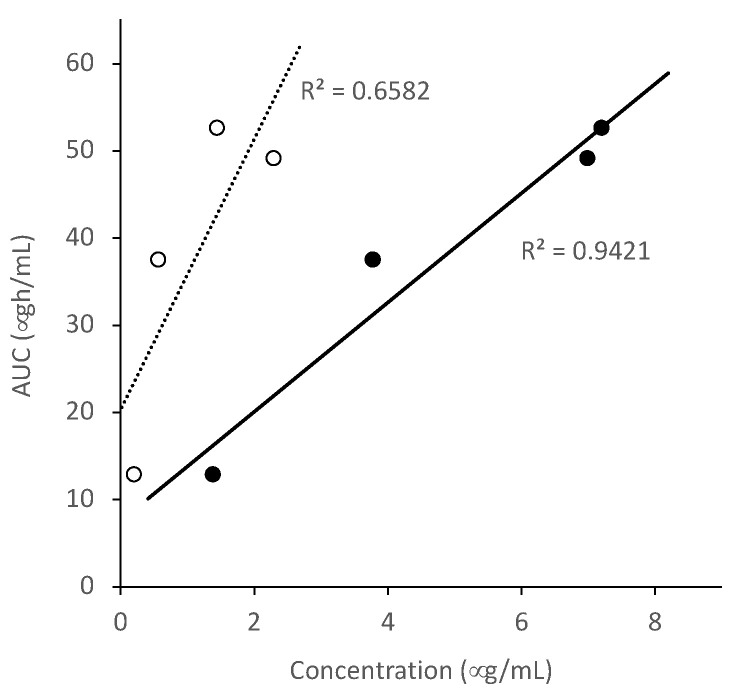
Relationship between final ALZ concentration in the μDiss dissolution study at pH 6.8 (open) or LLPS concentration (closed) and AUC in rats.

The absorption of ALZ from the ASDs was significantly higher than that from the crystalline ALZ in the rat study. The magnitude of effective improvement of the AUC and C_max_ by the polymers was in the following increasing order, PVPVA, HPMCAS, and Eudragit, which also agreed with that of the LLPS. The relationship between LLPS concentration and in vivo AUC is presented in Figure 8, where a good correlation was achieved. The relationship between the final ALZ concentration in the μDISS dissolution study in the JP2 solution (pH 6.8) indicated that the supersaturation behavior was elucidated successfully. However, its correlation with the AUC was much lower than that with LLPS concentration. The absorption of ALZ from the ASDs was likely limited by solubility because LLPS is analogous to amorphous solubility.

Most previously reported oral administration studies of ASDs have been performed with suspensions because of difficulty in administering solid dosage forms for small animals. However, ASDs are intended to be manufactured as solid dosage forms when they are marketed. Thus, the difference in the performance of suspensions and solid dosage forms has been increasingly observed recently. The most typical approach to fill this gap is to improve the dissolution behavior of ASDs using additives such as inorganic salts [21,22].

The oral absorption behavior in dogs was completely different from that in rats. In this study, ASDs were administered as solid dosage forms, which has more practical significance compared to the administration of suspensions. For connecting results from both animal species, the same HPMCAS ASD suspension was evaluated. Despite the large difference in physiologies between rats and dogs, a similar improvement in the oral absorption was confirmed using the HPMCAS ASD suspension. The capsule’s disintegration process appeared to dramatically affect the oral absorption, as evident from the large difference in absorption between the two HPMCAS ASDs (suspension and capsule). The absorption from the encapsulated solid ASD was comparable to that from the crystalline ALZ. The Eudragit ASD allowed better absorption than the other ASDs, even when it was administered as capsules, whereas absorption from the PVPVA ASD was worse than that from the crystalline ALZ. In this case, the absorption was not likely to have been governed by solubility but rather by the dissolution rate, and LLPS is not a predictor of oral absorption. The absorption profiles of ALZ from capsules were likely to be influenced by particle size of the recrystallized ALZ. That from the Eudragit ASD were permeable to some extent through the 0.2-μm filters (Figure 4b), suggesting that small ALZ particles should be available after the recrystallization. As for the HPMCAS ASD, the particle size analysis indicated that the ALZ particles’ mean size after the recrystallization was approximately 0.22 μm (Table 2). However, large ALZ crystals might be formed after the dissolution of the PVPVA ASD, which was likely to be the origin of the worse absorption than from crystalline ALZ. This analysis indicates that estimation of the particle size using filters with multiple pore sizes may explain oral absorption if it is limited by dissolution rate. However, for maximizing the efficacy of ASDs, the oral absorption should not be limited by dissolution rate but solubility. In this regard, PVPVA should have the highest potential to improve oral absorption of ALZ based on its highest LLPS concentration. Thus, after optimizing the composition of the binary ASDs, further formulation studies to decide the final dosage form are critical during the developmental study of ASDs. LLPS concentration should work as a parameter that can offer the potential for improvement of oral absorption.

## 5. Conclusions

ALZ was formulated into ASDs using three types of polymers: Eudragit, PVPVA, and HPMCAS, and their LLPS, dissolution, and oral absorption behaviors were investigated. The oral absorption in rats, where the ASDs were administered as suspensions, was not explained by the dissolution test but predicted by the LLPS concentration, which suggested the solubility-limited absorption of ALZ. The efficacy of the ASDs administered to dogs as solid capsules were likely to be limited by dissolution rate. Comprehension of particle size after LLPS appeared to be important for understanding the oral absorption in this case. This observation revealed the challenges in understanding supersaturation, LLPS, and crystallization behaviors occurring in the gastrointestinal tract for interpreting/predicting oral absorption. A formulation strategy that provides sufficiently fast disintegration and dissolution for achieving supersaturation was indicated to be important for ASDs.

## Figures and Tables

**Table 1:** Basic Properties of ALZ [16,17,18].

**Molecular Weight**		**pKa (Base), 25 °C**		**LogP, 25 °C**	
265.3		4.2		3.1	
**Solubility, 37 °C (μg/mL)**					
pH 1.2	FeSSIF_blk_ (pH 5.0)	FeSSIF (pH 5.0)	FaSSIF_blk_ (pH 6.5)	FaSSIF (pH 6.5)	pH 7.4
184	1.1	6.1	0.85	1.9	0.75

Subscript “blk” means the removal of taurocholic acid and lecithin from simulated intestinal fluids.

**Table 2:** Solution/suspension properties of ALZ in PB (pH7) in the presence and absence of polymers.

Polymers	No Polymer	Eudragit L100	HPMCAS	PVPVA
Solubility, 25 °C (μg/mL)	<0.10	0.44 ± 0.02	0.47 ± 0.07	<0.10
Solubility, 37 °C (μg/mL)	<0.10	0.32 ± 0.06	0.55 ± 0.09	<0.10
LLPS, 25 °C (μg/mL)	1.4	3.8	7.0	7.2
Particle size, 25 °C (μm)	>3	2.13 ± 0.23	0.22 ± 0.02	1.12 ± 0.06
Polydispersity Index (PDI)	−	0.59 ± 0.10	0.14 ± 0.02	0.21 ± 0.04
Zeta potential, 25 °C (mV)	−	−32.8 ± 1.2	−12.4 ± 1.1	−0.2 ± 0.2

**Table 3:** PK parameters for oral administration study of ALZ ASDs in rats.

Formulations	Crystalline ALZ	Eudragit ASD	HPMCAS ASD	PVPVA ASD
C_max_ (μg/mL)	2.17 ± 0.54	6.30 ± 1.14	8.06 ± 0.81	8.82 ± 1.44
T_max_ (h)	3.3 ± 1.2	4.0 ± 0.0	2.0 ± 0.0	2.3 ± 1.5
AUC (μg·hr/mL)	12.7 ± 3.5	37.4 ± 4.9	48.9 ± 5.4	52.4 ± 11.6

**Table 4:** PK parameters for oral administration study of ALZ ASDs in beagle dogs.

Formulations	Crystalline ALZ	Eudragit ASD	HPMCAS ASD	HPMCAS ASD Suspension	PVPVA ASD
C_max_ (μg/mL)	0.30 ± 0.21	0.44 ± 0.10	0.34 ± 0.09	0.57 ± 0.05	0.20 ± 0.07
T_max_ (h)	1.3 ± 0.6	1.7 ± 0.6	2.3 ± 1.5	1.3 ± 0.6	1.3 ± 0.6
AUC (μg·h/mL)	1.93 ± 1.27	2.81 ± 0.47	2.14 ± 0.42	3.04 ± 0.13	1.04 ± 0.41

## Data Availability

Detailed data presented in this study are available in supplementary material.

## Supplementary Materials

The following are available online at https://www.mdpi.com/1999-4923/13/2/220/s1, Figure S1. TG-DTA and Polarized microscopy data for ASDs presented in this study. (a): HPMCAS ASD; (b): PVPVA ASD (3:1); (c): PVPVA ASD (4:1); (d): Eudragit ASD; Table S1. Numerical data for the LLPS dissolution test (Figure 4); Table S2. Numerical data for the rat study (Figure 6); Table S3. Numerical data for dog study (Figure 7).
