# Supplementary material for: Relevance of Liquid-Liquid Phase Separation of Supersaturated Solution in Oral Absorption of Albendazole from Amorphous Solid Dispersions
Source: Pharmaceutics. 2021 Feb 5;13(2):220. doi: 10.3390/pharmaceutics13020220 (PMC7914868; doi:10.3390/pharmaceutics13020220)
Supplement: Supplementary file 1 [file pharmaceutics-13-00220-s001.pdf]

pharmaceutics

Supplementary Materials: Relevance of Liquid-Liquid Phase Separation of Supersaturated Solution in Oral Absorption of Albendazole from Amorphous Solid Dispersions

Kyosuke Suzuki, Kohsaku Kawakami, Masafumi Fukiage, Michinori Oikawa, Yohei Nishida, Maki Matsuda and Takuya Fujita

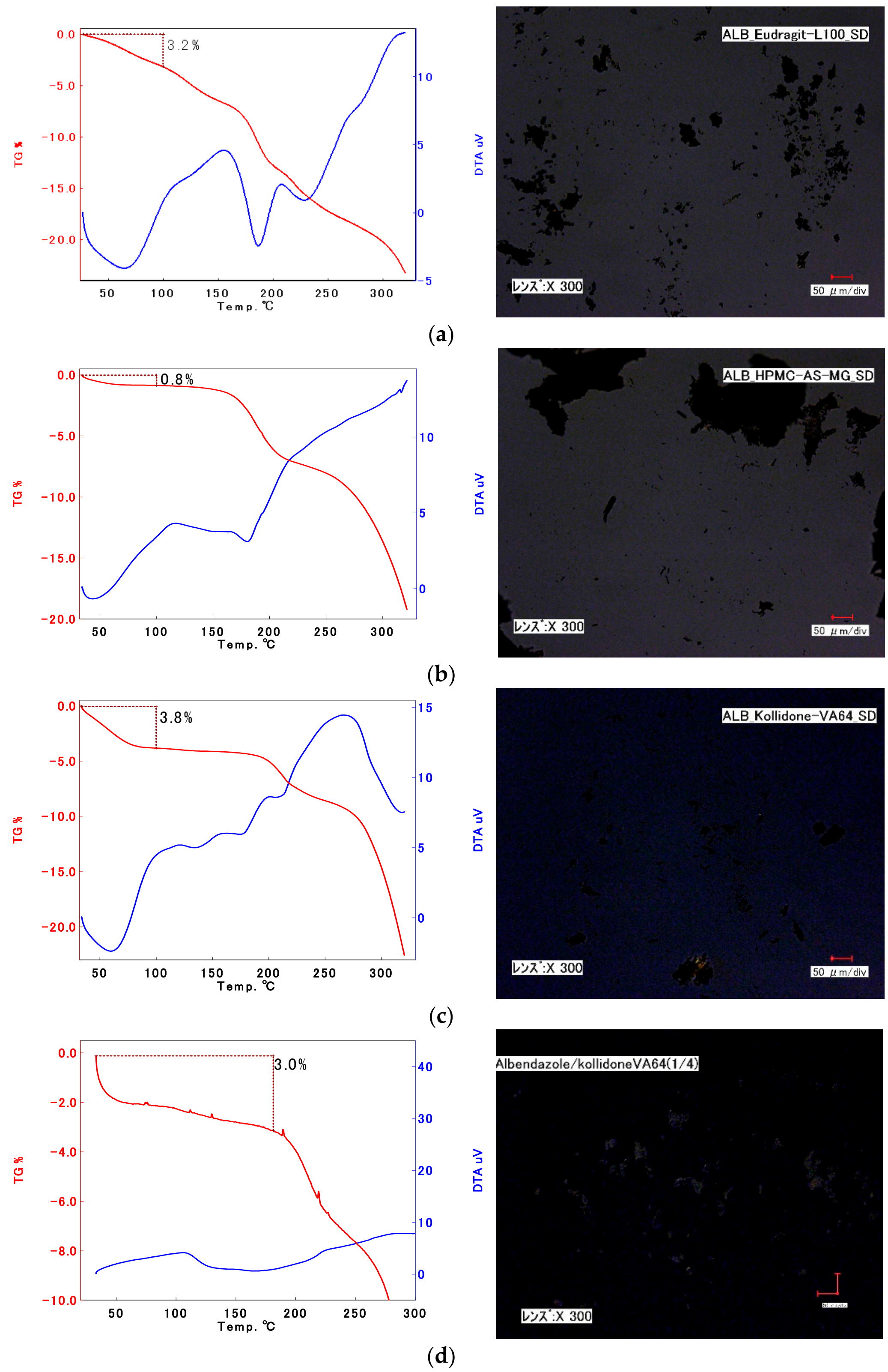

Figure S1. TG-DTA and Polarized microscopy data for ASDs presented in this study. (**a**): HPMCAS ASD; (**b**): PVPVA ASD (3:1); (**c**): PVPVA ASD (4:1); (**d**): Eudragit ASD.

Table S1. Numerical data for the LLPS dissolution test (Figure 4).

	0.2 um				0.7 um			
Time (min)	Crystal	PVPVA	Eudragit	HPMCAS	Crystal	PVPVA	Eudragit	HPMCAS
0	0	0	0	0	0	0	0	0
1	0.000	0.223	1.705	0.197	0.002	2.157	2.164	1.838
3	0.015	0.303	1.923	0.454	0.045	2.432	3.082	2.317
5	0.063	0.359	2.017	0.299	0.089	2.526	3.350	2.374
10	0.087	0.382	2.082	0.339	0.107	2.839	3.778	2.477
20	0.093	0.410	2.174	0.367	0.146	2.750	3.561	2.522
30	0.105	0.433	2.221	0.369	0.144	2.303	3.074	2.501

Table S2. Numerical data for the rat study (Figure 6).

Time after administration (hr)	Crystalline ALZ	SD		PVPVA ASD	SD
0	0	0		0	0
0.25	0.52	0.04		3.35	0.67
0.5	1.08	0.16		5.10	0.82
1	1.81	0.08		7.31	0.20
2	2.17	0.27		8.82	1.69
4	2.17	0.78		8.09	2.50
8	0.52	0.38		3.33	1.35
	HPMCAS ASD	SD		Eudragit ASD	SD
0	0	0		0	0
0.25	2.69	0.35		1.10	0.31
0.5	4.38	0.22		2.21	0.61
1	6.78	0.54		3.99	0.69
2	8.06	0.81		5.77	0.93
4	7.61	0.87		6.30	1.14
8	3.29	1.19		2.90	1.18

Table S3. Numerical data for dog study (Figure 7).

Time (hr)	Crystal SD	SD	PVPVA	SD	Eudragit	SD
0	0.0	0.0	0.0	0.0	0.0	0.0
0.25	6.1	6.9	51.8	58.4	51.8	36.2
0.5	117.1	70.7	123.3	82.4	265.0	61.5
1	253.8	136.5	195.4	75.0	429.8	82.4
2	303.2	217.8	188.9	71.7	435.1	102.3
4	272.9	228.7	119.3	41.5	406.1	94.8
7	211.9	175.6	97.8	68.5	252.3	28.0
Time (hr)	HPMCAS	SD	HPMCAS susp	SD		
0	0.0	0.0	0.0	0.0		
0.25	127.5	105.7	271.1	58.4		
0.5	187.4	135.0	468.2	19.4		
1	267.3	158.9	538.9	133.7		
2	339.6	86.7	565.3	75.6		
4	301.6	45.6	338.1	28.6		
7	212.8	12.5	265.6	16.9		
